# Reconstruction of the pelvis after traumatically induced bilateral partial hemipelvectomy: a case report

**DOI:** 10.1186/s13256-019-2283-5

**Published:** 2019-12-04

**Authors:** Ayako Kamitomo, Minoru Hayashi, Ryohei Tokunaka, Yuki Yoshida, Sayo Tatsuta, Yoshie Sasaki

**Affiliations:** Department of Plastic, Reconstructive and Aesthetic Surgery, Japan Red Cross Maebashi Hospital, 389-1 Asakura-cho, Maebashi-shi, Gunma 3710811 Japan

**Keywords:** Traumatic hemipelvectomy, Transcatheter arterial embolization, Amputation, Reconstruction, Anterolateral thigh flap

## Abstract

**Background:**

Traumatic hemipelvectomy is a catastrophic fracture of the pelvis as a result of high-energy trauma, such as in a car accident. There have been few case reports of traumatic hemipelvectomy because many of these patients die before they are transferred to a hospital. However, an increasing number of patients are being saved and admitted to hospital due to improvements in resuscitation and the emergency response system. Accordingly, there has been a growing body of reports on the management and reconstruction of traumatic hemipelvectomy.

**Case presentation:**

A healthy 20-year-old Japanese man was trapped beneath a 3-ton steel frame while working on a crane. We describe here a very challenging case of traumatically induced bilateral partial hemipelvectomy with successful reconstruction of our patient’s pelvis using a unilateral anterolateral thigh flap.

**Conclusion:**

To the best of our knowledge, there have been few reports of bilateral hemipelvectomy and our case is the first to be successfully treated with a unilateral anterolateral thigh flap.

## Introduction

Traumatic hemipelvectomy (TH) is a rare catastrophic fracture of the pelvis as a result of high-energy trauma, such as in a car accident. Some cases of TH have been reported, most of which were unilateral hemipelvectomy. We present here a very challenging case of traumatically induced bilateral partial hemipelvectomy with successful reconstruction of the pelvis using a unilateral anterolateral thigh flap.

## Case presentation

**Fig. 1:**
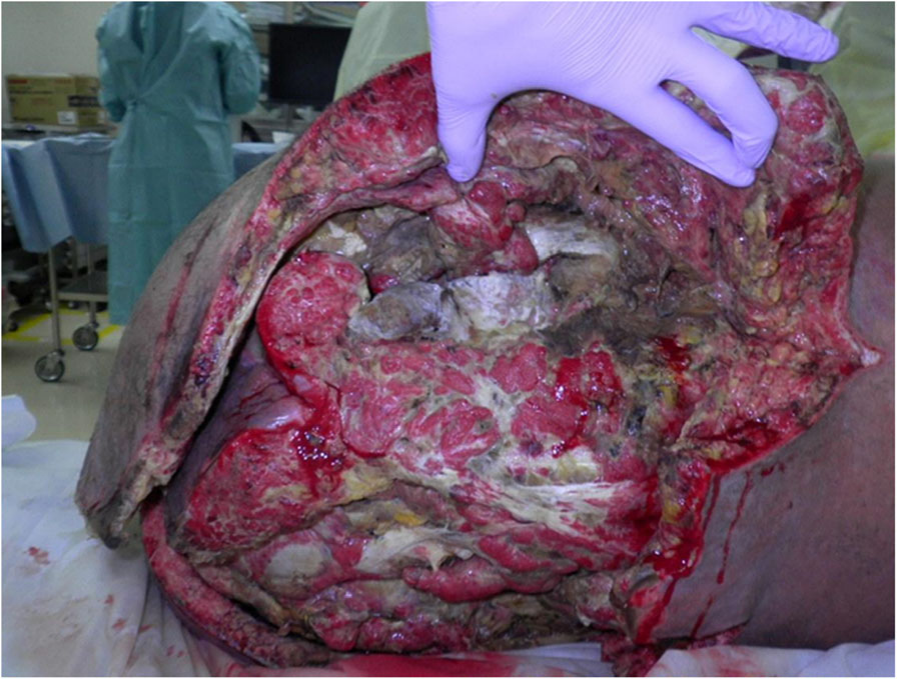
Before the debridement

**Fig. 2:**
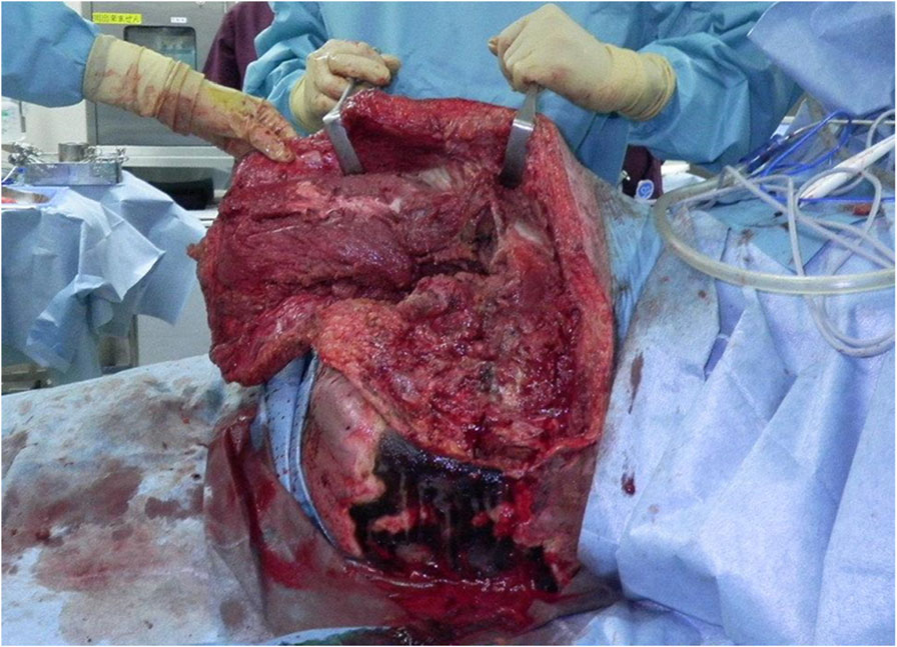
After the debridement

**Fig. 3:**
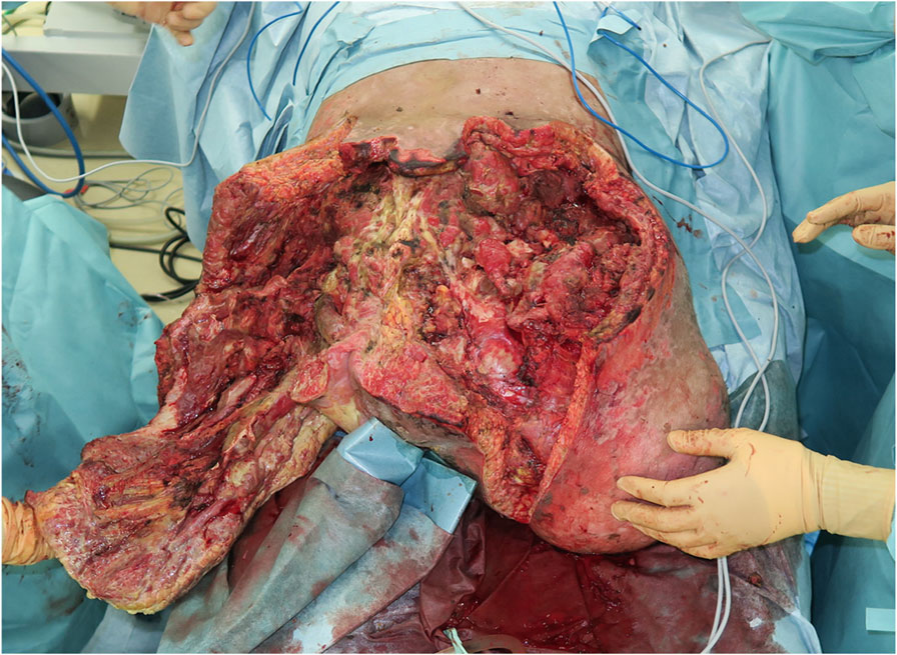
Left anterolateral thigh flap was preserved after several debridements

**Fig. 4:**
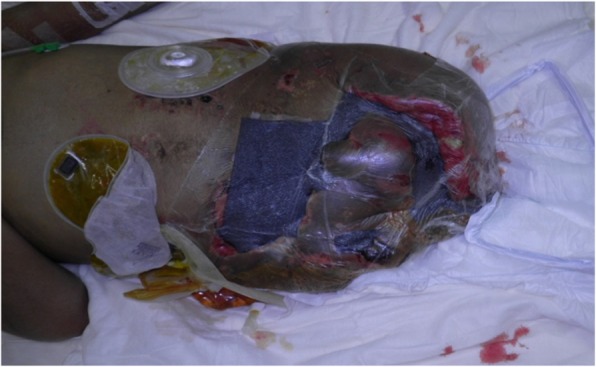
Negative-pressure wound therapy was applied

**Fig. 5:**
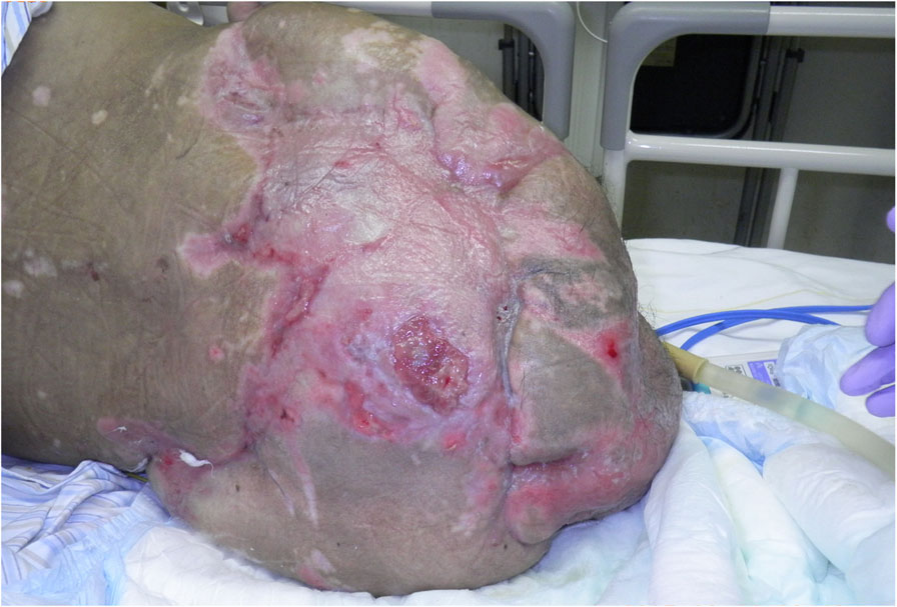
In lateral position. Epithelialization was achieved

**Fig. 6:**
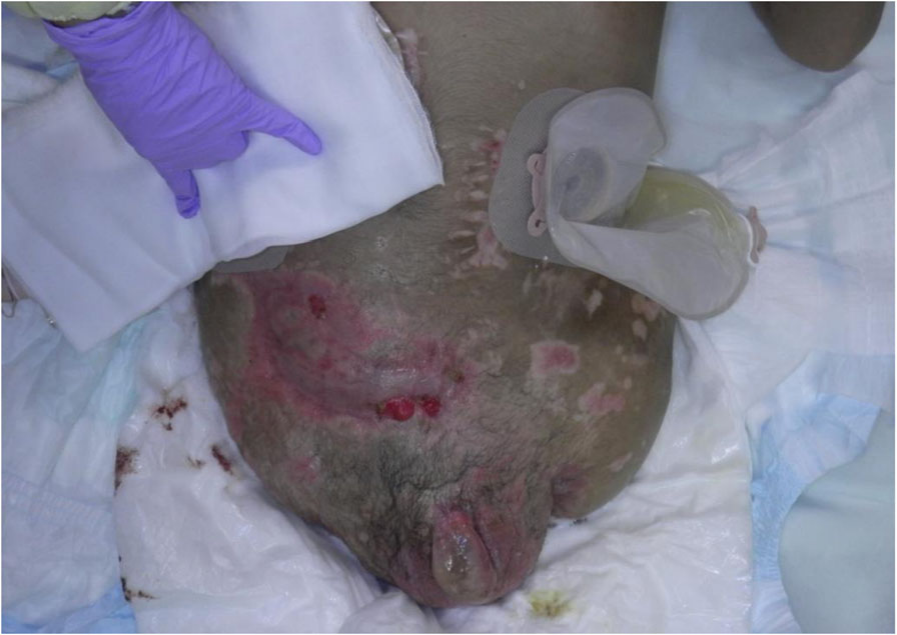
In supine position. Epithelialization was achieved

**Fig. 7:**
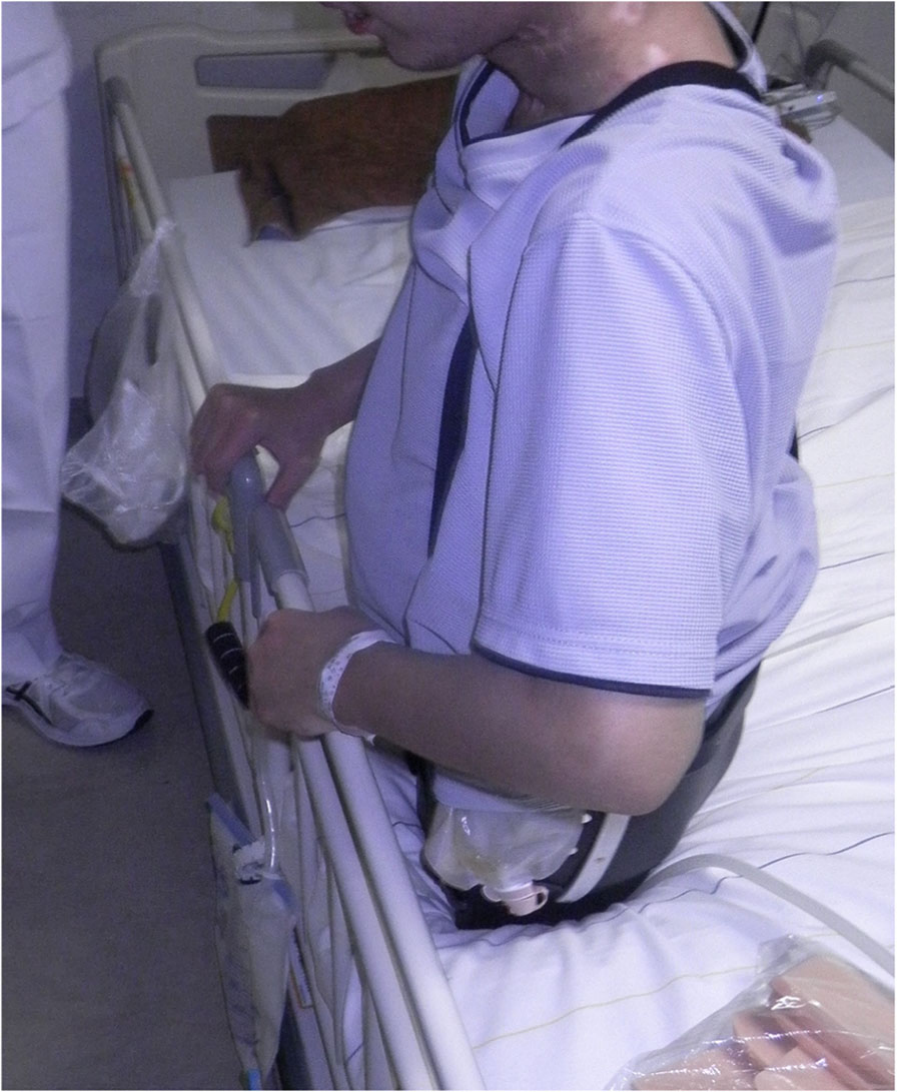
Rehabilitation was started along with the use of prosthetics

A healthy 20-year-old Japanese man was trapped beneath a 3-ton steel frame while working on a crane. He was rescued by his coworkers and exhibited constant bleeding from the right side of his lower abdomen when the ambulance arrived. He was transferred to our emergency department with the wound compressed. On arrival, his vital signs were: respiratory rate 22/minute, blood pressure 97/65 mmHg, and pulse rate 140/minute. His Glasgow Coma Scale score was E4 V5 M6. On physical examination, there was instability of his hip and constant bleeding from the wound on the right side of his lower abdomen. Endotracheal intubation and fluid resuscitation were performed. Computed tomography (CT) of his body showed: splenic injury; sacrum fracture; separation of the sacroiliac joint; and fracture of the right pubis, ischium, and acetabulum. Vancomycin and meropenem were started to prevent infection. He was immediately taken to our angiography room for transcatheter arterial embolization (TAE) for pelvic hemorrhage. Angiography showed extravasation from the bilateral internal iliac arteries, which were embolized. His bilateral external iliac arteries showed thrombosis. A SAM® Pelvic Sling™ (SAM Medical, Wilsonville, OR, USA) was applied to his pelvis. Since complete hemostasis was not achieved after TAE, a vascular surgeon was consulted. Based on the view from the expanded wound on the right side of his lower abdomen, his right external artery was ligated in the emergency room and hemostasis was achieved. Gauze was packed with the wound sutured. Subsequent CT showed ischemia of his right lower limb. Despite the amount of time that had passed since the accident and the possibility of increased risk, due to his young age and the strong desire of his family to save his limbs, revascularization surgery was performed in the operating room. After crossover graft of the axillary-femoral artery, another hemorrhage was confirmed in his retroperitoneum, which was thought to be due to the external iliac artery injury. An additional crossover graft of the axillary-femoral vein was performed. Cystostomy and external fixation of the pelvic fracture were performed. On day 2, additional debridement of the right side of his lower abdomen was performed. Packed gauze was removed and a drainage tube was inserted. Contrast CT showed ischemia of his left lower limb and right anterior tibial artery. On day 3, he was referred to our plastic surgery department for blisters on his bilateral thighs and scrotum, which were thought to be a complication of TAE of his bilateral internal iliac arteries. At a conference meeting with the intensive care unit team and orthopedic surgery team, we concluded that amputation of his bilateral lower limbs was inevitable, since we prioritized saving life over limb. On day 8, a right above-knee amputation was performed. The decision to amputate above the knee was made because there was a possibility that the soft tissue could be covered with the intact skin of his thigh. There was also a risk of massive hemorrhage due to the pelvic fracture if we performed hip disarticulation. On day 13, left hip disarticulation and colostomy were performed. On day 17, bilateral ureterostomy was performed. On day 21, he underwent another debridement. We confirmed necrosis in his bilateral gluteal muscles and part of his quadriceps muscle, which were debrided (Fig. 1). Because it was difficult to control the hemorrhage, we had to stop debridement, even though necrotic muscle was still visible. We left the wound open because of the extensive soft tissue damage (Fig. 2). Repeated debridement was necessary because of the ongoing infection; we performed debridement a total of six times (Fig. 3). Part of his ilium and acetabulum was left and the posterior ilium was fully exposed after the extensive debridement. On day 112, left anterolateral thigh flap was rotated posteriorly and fixed with a suture anchor with successful coverage of the exposed pelvis. Negative-pressure wound therapy (NPWT) was applied for the formation of healthy granulation tissue (Fig. 4). On day 168, a full-thickness skin graft, which was harvested from his head, back, and abdomen, was applied to the rest of the ulcer. On day 230, complete epithelialization was achieved (Figs. 5, 6). Rehabilitation was started along with the use of prosthetics (Fig. 7). He has made progress to the point where he can get on and off a wheelchair and move around independently. Currently, he lives at home with his parents. The reconstructed pelvis allows for good prosthetic fitting.

## Discussion

TH is a severe form of pelvic fracture that accounts for 0.55–2% of all cases of pelvic fracture [1–4]. It is defined as an open or closed complete dislocation of the hemipelvis with disruption through the symphysis pubis and sacroiliac joint combined with traumatic rupture of the iliac vessels [1]. Out of all of these patients, 50% present with the limb attached to the body [2].

Pelvic fracture in general is a catastrophic injury with a mortality rate of 40% and this percentage rises to 60 to 100% in patients with TH [3, 5]. Immediate management of the hemorrhage is the top priority. In this case, immediately after hemostatic resuscitation was performed, angiography was performed for TAE to control the continuous hemorrhage, and the bilateral internal iliac arteries were embolized. TAE has been shown to be useful for controlling arterial hemorrhage and is often successfully performed for hemorrhage control in cases of pelvic fracture. In this case, the hemorrhage was also due to injury of the right external iliac artery, which was successfully treated by ligation. In parallel with the increasing number of TAE procedures performed for pelvic fracture, there has been an increase in the number of complications caused by TAE of the internal iliac artery, including gluteal skin and muscle necrosis, dysfunction of the bladder and rectum, and sexual dysfunction. Our patient was referred to our plastic surgery department due to the presence of blisters on his bilateral thighs and scrotum, which were thought to be complications caused by TAE of the bilateral internal arteries. These blisters were evident at our first consultation. In addition, his limbs were pale and contrast CT showed ischemia of his lower limbs. Rapid amputation of his bilateral limbs was inevitable to prevent the progression of necrosis and infection. The bilateral limbs were amputated in separate procedures because the amount of blood lost in each operation could have easily exceeded 2000 ml and our patient could not have tolerated such a prolonged surgery. We needed to perform several debridement procedures because of the massive hemorrhage, which placed a tremendous burden on our patient; the amount of nonviable tissue that needs to be debrided tends to be underestimated since it is very difficult to differentiate between viable and nonviable tissue. Gelatin sponge, which was used for embolization of his bilateral internal iliac arteries, was reperfused 2 or 3 days after embolization and the blood supply was maintained. This could also explain why tissue and skin that appeared to be viable during debridement often turned necrotic several days later.

Reconstruction after bilateral partial hemipelvectomy is very important for rehabilitation. The dead space of the pelvis needs to be filled with an adequate amount of soft tissue. A simple skin graft cannot tolerate the shear force and could easily lead to the formation of ulcers. Of the methods that have been reported regarding soft tissue coverage after hemipelvectomy, most involve hemipelvectomy following osteomyelitis or the removal of malignancies [6–8]. In a situation where a tumor in the pelvis is removed, intact skin and muscle can be preserved to some extent, and the choice of a free flap from the ipsilateral leg could also be considered. Besides the traditional posterior gluteus maximus flap or anterior quadriceps flap reconstruction after hemipelvectomy, Senchenkov *et al*. reported that the rectus abdominis muscle or musculocutaneous flap is recommended for large defects after the removal of cancer [6]. Marfori and Wang reported the use of adductor myocutaneous flap coverage for hemipelvectomy after the removal of cancer [7]. McKnight *et al*. reported using femur, fibula, and fillet of leg flaps from the amputated extremity [8]. Faria *et al*. reported three cases of fillet flap reconstruction after hemipelvectomy [9]. However, in the case of TH, a standard flap, such as a gluteal flap and a free flap from the ipsilateral or contralateral leg, cannot be relied upon due to extensive skin and soft tissue damage, as in our case. Some reconstruction methods after traumatic unilateral hemipelvectomy have been reported. Kayalar *et al*. used a posterior flap to cover the defect [3]. Timmers *et al*. used an anterolateral thigh flap from the contralateral leg [1]. Purcell *et al*. reported the first reconstruction using a residual hamstring rotational flap [10]. To the best of our knowledge, most of these reports involved reconstruction after unilateral hemipelvectomy. We found one case of bilateral traumatically induced hemipelvectomy, but it did not provide a detailed discussion about reconstruction [11]. In our case, we tried to preserve intact soft tissue and skin as much as possible under the consideration that exposed remaining partial bilateral pelvic bone needed to be covered with ample soft tissue. We also tried to preserve our patient’s pelvis because it would play an important role in bearing his weight and maintaining balance with a prosthetic fitting. His remaining bilateral pelvis after bilateral partial hemipelvectomy was asymmetric and therefore enough soft tissue was needed to be able to tolerate the shear force of sitting or standing while wearing a prosthesis. We considered using a free latissimus dorsi flap; however, we wanted to preserve them because our patient would need strength to hold his body and use a wheelchair. During treatment, we applied NPWT to the open wound of his pelvis to decrease the defect in as short a time as possible. We performed reconstruction of his pelvis using a unilateral anterolateral thigh muscle and continued the application of NPWT to the soft tissue until the final skin graft was performed on day 168. Complete epithelialization of our patient’s pelvis was achieved on day 230 and rehabilitation was started along with the creation of a pelvic prosthesis socket.

Despite his catastrophic injuries, he has shown remarkable progress with his rehabilitation along with management of his mental health through early intervention by a psychiatric team. His young age and his potential physical ability have enabled him to rapidly adjust to his new situation. The support and understanding of his family and his surroundings also contributed to his recovery.

## Conclusion

We described a challenging case of a 20-year-old man with traumatically induced bilateral hemipelvectomy with successful reconstruction of the bilateral pelvis using a unilateral anterolateral thigh flap. Aggressive resuscitation and early hemorrhage control contributed to his survival. Early intervention by a plastic surgery team is important when planning the method that will be used for reconstruction of the pelvis. In this case, a multidisciplinary approach that included an emergency physician, orthopedist, vascular surgeon, psychiatrist, physiatrist, general surgeon, urologist, and anesthesiologist was needed for successful management of such a catastrophic injury.

## Data Availability

Not applicable.
